# Antagonistic Polyphenol Interactions Underlie the α-Glucosidase Inhibitory Activity of Keemun Black Tea

**DOI:** 10.3390/foods15061061

**Published:** 2026-03-18

**Authors:** Xiao-Lan Yu, Xizhe Zhu, Xinxin Lv, Jingming Ning, Haibo Yuan

**Affiliations:** 1 Tea Research Institute, Chinese Academy of Agricultural Sciences, Hangzhou 310008, China; yuxiaolan@tricaas.com (X.-L.Y.); transam3870@gmail.com (X.Z.); 2 School of Tea Science, Anhui Agricultural University, 130 Changjiang West Road, Hefei 230036, China; l2082372843@163.com

**Keywords:** Keemun black tea, esterified catechins, theaflavins, α-glucosidase inhibition, antagonism, reconstruction–omission, combination index

## Abstract

The α-glucosidase inhibitory activity of Keemun black tea arises from complex interactions among its major polyphenols, which cannot be reliably predicted from the activities of isolated compounds. In this study, eight dominant polyphenols were investigated using a quantitative reconstruction–omission framework designed to reflect typical household tea brewing. The fully reconstructed system recovered approximately 72% of the inhibitory activity of the diluted native infusion, supporting the functional representativeness of the selected compounds. Systematic omission experiments revealed that antagonistic interactions, particularly among theaflavins, dominated the net inhibitory outcome, with removal of the most potent inhibitor, theaflavin-3,3′-digallate (TFDG), paradoxically increasing overall activity. Pairwise Combination Index analysis further demonstrated concentration-dependent biphasic interactions, exemplified by the epigallocatechin-3-gallate (EGCG)–TFDG pair, while molecular docking suggested overlapping binding sites as a potential structural basis for competitive inhibition. Collectively, this work provides a system-level dissection of α-glucosidase inhibition in black tea. Although the reconstructed system does not fully capture all contributions, the proposed framework offers a generalizable strategy for investigating interaction-driven bioactivity in complex dietary matrices and for further mechanistic studies.

## 1. Introduction

Type 2 diabetes mellitus (T2DM) is a major global health challenge, accounting for over 90% of all diabetes cases [1,2]. Postprandial hyperglycemia is a key driver of T2DM progression [3], making inhibition of α-glucosidase—the primary intestinal enzyme responsible for carbohydrate digestion—a critical therapeutic target [4]. Although synthetic inhibitors such as acarbose are effective, their use is often limited by gastrointestinal side effects [5], motivating the search for safer, diet-derived alternatives. Black tea, one of the most widely consumed beverages worldwide, consistently exhibits α-glucosidase inhibitory activity in vitro, in vivo, and in clinical trials [6,7,8]. This bioactivity is largely attributed to its distinctive polyphenol composition formed during fermentation, in which flavan-3-ols such as (-)-epigallocatechin-3-gallate (EGCG) and (-)-epicatechin gallate (ECG) undergo enzymatic oxidation and dimerization to produce theaflavins, including theaflavin (TF), theaflavin-3-gallate (TF3G), theaflavin-3′-gallate (TF3′G), and theaflavin-3,3′-digallate (TFDG) [9,10,11].

Extensive studies have demonstrated that individual tea polyphenols—including esterified catechins such as EGCG, ECG, (–)-gallocatechin gallate (GCG), and (–)-catechin gallate (CG), as well as theaflavins—exhibit strong α-glucosidase inhibitory activity when evaluated as isolated compounds [11,12]. Nevertheless, how these polyphenols act collectively within brewed tea—and whether their combined effects can be predicted by simple additivity—remains unclear. In realistic food matrices, polyphenols coexist at varying concentrations, where interaction-driven effects [13,14,15] may render reductionist predictions unreliable. Consequently, studies focusing solely on isolated compounds or simplified binary pairs at non-consumption-relevant ratios provide limited insight into the functional behavior of brewed tea. These interaction-driven effects are critical to consider, because the behavior of individual polyphenols in isolation may not reliably reflect their functional contributions in the full tea matrix. Compounds with strong inhibitory activity on their own can exhibit effects that deviate from predictions based on isolated-compound behavior when coexisting with other polyphenols under consumption-relevant conditions. Such behavior underscores the importance of evaluating bioactivity within chemically realistic systems that preserve both compositional complexity and relative abundance.

Although black tea contains a wide range of phenolic constituents, the present study focuses on eight dominant polyphenols selected based on a dual functional–compositional rationale. These compounds—four esterified catechins (EGCG, ECG, GCG, and CG) and four theaflavins (TF, TF3G, TF3′G, and TFDG)—are consistently reported as the major, well-characterized polyphenols in black tea infusions [16,17] and exhibit clear and significant α-glucosidase inhibitory activity. Rather than aiming to exhaustively represent the entire phenolic profile, this study focuses on reconstructing a functionally dominant core module that captures the principal contributors to α-glucosidase inhibition under consumption-relevant conditions reflecting typical household tea brewing. The adequacy of this selection is subsequently evaluated by assessing the extent to which the reconstructed system recovers the inhibitory activity of the native tea infusion.

Taken together, the above considerations indicate that understanding α-glucosidase inhibition in brewed tea requires an experimental strategy capable of resolving both individual contributions and interaction-dependent effects within a chemically realistic system. Building on this premise, this study hypothesizes that the net α-glucosidase inhibitory activity of black tea is governed by antagonistic and synergistic interactions among its major polyphenols, rather than by individual compounds alone. To evaluate this hypothesis, a quantitative reconstruction–omission framework—in which the overall activity of a complex system is systematically reconstructed from, and selectively perturbed by omitting, individual components—a strategy previously employed to dissect flavor and sensory attributes in tea [18,19,20,21], was applied. Compared with conventional reductionist designs, this framework enables systematic attribution of net functional contributions while maintaining the relative proportions and concentration relationships among coexisting polyphenols characteristic of brewed tea. Importantly, the application of this system-level approach to α-glucosidase inhibitory activity has not been reported, representing an unexplored extension of the method beyond sensory evaluation.

The main objective of this work is to elucidate how major polyphenols collectively govern α-glucosidase inhibition in Keemun black tea—one of the representative high-aroma black teas—employed in this study as a model system under consumption-relevant conditions. Specifically, this study (i) reconstructed the full polyphenol system to evaluate its fidelity in recovering the inhibitory activity of the native infusion; (ii) performed systematic omission experiments to determine the net contributions of individual polyphenols and their interactions; (iii) applied pairwise Combination Index (CI) analysis to quantify synergistic or antagonistic effects; and (iv) conducted molecular docking to explore potential structural correlates of synergistic or antagonistic interactions.

By integrating chemical, functional, and structural analyses, this study provides a system-level understanding of how polyphenol interplay shapes α-glucosidase inhibition in black tea and establishes a generalizable framework for investigating bioactivity in complex dietary matrices.

## 2. Materials and Methods

### 2.1. Chemicals and Reagents

Chromatographic standards of CG, GCG, ECG, and EGCG (≥98% purity) were purchased from Sichuan Weikeqi Biological Technology Co., Ltd. (Chengdu, China), Shanghai Aladdin Biochemical Technology Co., Ltd., and Shanghai Macklin Biochemical Technology Co., Ltd. (Shanghai, China). Theaflavins (TF ≥ 95% purity; TF3G, TF3′G, and TFDG ≥ 98% purity) were obtained from Shanghai Yuanye Biological Technology Co., Ltd. (Shanghai, China).

α-Glucosidase (from *Saccharomyces cerevisiae*, EC 3.2.1.20, 25 U/mg solid), p-nitrophenyl-α-D-glucopyranoside (pNPG, ≥99%), and acarbose (≥95%) were also supplied by Shanghai Yuanye Biological Technology Co., Ltd. (Shanghai, China). Keemun black tea (cultivar *Zhu Ye Zhong*, harvested in Qimen County, Anhui Province) was provided by Xiamen Qichun Tea Co., Ltd. (Fujian, China). All other reagents were of analytical grade.

### 2.2. Tea Infusion Preparation and Polyphenol Quantification

#### 2.2.1. Preparation of Tea Infusion

Tea infusion was prepared under consumption-relevant conditions following the National Standard of the People’s Republic of China [22]. Briefly, 3.0 g of Keemun black tea leaves were steeped in 150 mL of boiling deionized water (100 °C) for 5 min. The infusion was filtered immediately after brewing.

For enzymatic assays, the tea infusion was serially diluted with phosphate buffer to generate concentration–response curves for IC_50_ determination. Based on preliminary dose–response curves, a 100-fold dilution of the infusion was selected for reconstruction and interaction experiments, yielding 70–80% inhibition, which falls within the dynamic portion of the IC_50_ curve—avoiding both insensitivity at low inhibition and plateauing near complete inhibition. Concentrations of the eight target polyphenols in this diluted infusion served as the reference for the reconstruction mixtures.

#### 2.2.2. HPLC Analysis of Catechins and Theaflavins

Polyphenol profiles were determined using an Agilent 1100VL HPLC system (Agilent Technologies Inc., Santa Clara, CA, USA) equipped with a variable wavelength detector (VWD). Separation and quantification followed established protocols [23,24] with minor modifications. Samples were filtered through a 0.22 μm membrane prior to analysis.

For catechins, separation was performed on a C18 column (5 μm, 4.6 mm × 250 mm; Waters Corp., Milford, MA, USA) maintained at 35 °C. Mobile phases consisted of 2% (*v*/*v*) acetic acid in water (A) and acetonitrile (B) at a flow rate of 1.0 mL/min, using the following gradient: 0–16 min, 6.5–15% B; 16–25 min, 15–25% B; 25–25.5 min, 25–6.5% B; 25.5–30 min, 6.5% B. Detection was conducted at 280 nm.

For theaflavins, the flow rate was 1.5 mL/min with a gradient of 0–35 min, 20–25% B; 35–38 min, 25% B; 38–40 min, 25–20% B, with detection at 380 nm. Individual polyphenols were quantified using the external standard method [23,25,26].

### 2.3. α-Glucosidase Inhibition Assay and IC_50_ Determination

The assay was conducted as previously described [27] with minor modifications. For IC_50_ measurements, serial dilutions of the tea infusion or individual polyphenol standards were prepared in 0.1 M phosphate buffer (pH 6.8). Polyphenol standards were initially dissolved in dimethyl sulfoxide (DMSO), and subsequent dilutions ensured that the final DMSO concentration in the assay was ≤0.5% (*v*/*v*), a level confirmed not to affect α-glucosidase activity.

Briefly, 50 μL of sample solution and 100 μL of α-glucosidase (1 U/mL from *S. cerevisiae*) were pre-incubated at 37 °C for 10 min in a 96-well plate. The reaction was initiated by adding 50 μL of *p*NPG solution (5 mM) and maintained at 37 °C for 5 min. Absorbance was measured at 405 nm using a ReadMax 500F microplate reader (Shanghai Flash Spectrum Biological Technology Co., Ltd., Shanghai, China).

The control consisted of enzyme and substrate incubated with buffer containing the same volume of solvent used for sample preparation but without inhibitors. Blanks without enzyme were included to correct background absorbance. Inhibition rate (%) was calculated as follows:

(1) $$Inhibition\;\left(\%\right)=\left(1-\frac{A_{sample\;after}-A_{sample\;before}}{A_{control\;after}-A_{control\;before}}\right)\times 100\%$$

IC_50_ values were obtained by fitting dose–response curves to a four-parameter logistic (4PL) model using OriginPro 2025b (OriginLab Corp., Northampton, MA, USA). Acarbose served as a positive control. Although *S. cerevisiae* α-glucosidase differs from mammalian enzymes, it provides a robust model for screening inhibitory trends and interaction effects [6,27]. However, absolute inhibitory values may not directly translate to mammalian α-glucosidases due to structural and kinetic differences; therefore, the present results primarily reflect relative interaction patterns rather than precise in vivo potency.

All polyphenol solutions and dilutions were freshly prepared prior to assays to minimize degradation, and incubation time was kept short and consistent across experiments.

### 2.4. Reconstruction and Omission Design

A full reconstruction mixture was prepared by combining the eight selected polyphenol standards at concentrations equivalent to those measured in the 100-fold diluted tea infusion. Reconstruction mixtures were freshly prepared immediately prior to enzymatic assays.

**Figure 1:**
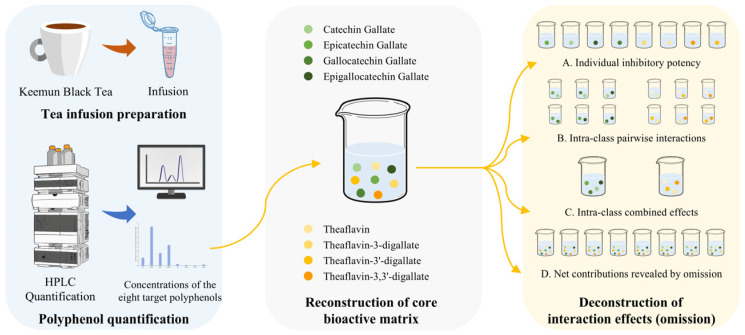
Schematic overview of the reconstruction–omission experimental strategy.

As illustrated in Figure 1, omission groups were generated by selectively removing individual compounds or predefined subclasses while maintaining all remaining components at their baseline concentrations. Experimental inhibition rates of reconstructed and omission mixtures were compared with those of the native infusion to assess the fidelity of the reconstruction.

Theoretical additive values were calculated as the arithmetic sum of the inhibition rates of the individual polyphenols at the same concentrations used in the mixture, assuming no interaction between compounds. Deviations of experimental inhibition from these additive values were interpreted as indicative of synergistic or antagonistic interactions.

### 2.5. Combination Index (CI) Analysis

To quantitatively evaluate interaction effects between polyphenols, the Chou-Talalay method [28] was employed. This approach is based on the median-effect equation, derived from the mass-action law principle, enabling quantitative comparison of single compounds and their combinations across a defined effect range. Under this framework, the resulting combination index (CI) [28,29] value of Chou-Talalay provides a quantitative definition of additive effect (CI = 1), synergism (CI < 1), and antagonism (CI > 1) in combinations.

Importantly, CI analysis captures interaction behavior as a function of concentration and effect level, allowing identification of biphasic or concentration-dependent interaction patterns. In complex food-derived systems, this dose–effect-based framework enables the identification of interaction-driven deviations from additivity without presupposing specific molecular mechanisms.

For each polyphenol pair, the concentration ranges tested were defined based on their respective IC_50_ values and covered low to high effect levels. Specifically, pairs between EGCG and individual TFs were tested from 0.39 to 12.5 μM. CI values of selected polyphenol pairs were calculated based on the mean inhibition rates of three independent measurements using CompuSyn software (Version 1.0, CompuSyn, Inc., Paramus, NJ, USA).

### 2.6. Molecular Docking

Molecular docking was conducted following reported protocols [30,31,32]. The crystal structure of *S. cerevisiae* α-glucosidase (PDB ID: 3A4A) was obtained from the RCSB Protein Data Bank. Ligand structures were retrieved from PubChem and converted to three-dimensional conformations using SwissParam with MMFF-based geometry optimization.

Docking simulations were performed using the CB-Dock2 platform, which integrates cavity detection with AutoDock Vina (1.2.0)-based flexible docking. Prior to docking, bound ligands and non-essential ions were removed from the protein structure. Docking poses were visualized using PyMOL (Version 3.0, Schrödinger, LLC. New York, NY, USA), with interacting residues defined as those within 4.0 Å of the ligand.

### 2.7. Statistical Analysis

All experiments were conducted in triplicate, and results are expressed as mean ± standard deviation. Statistical significance was evaluated using one-way analysis of variance (ANOVA) followed by Tukey’s post hoc test (*p* < 0.05) in OriginPro 2025b.

## 3. Results and Discussion

### 3.1. Single-Compound Activity and System Reconstruction

The absolute concentrations of the eight dominant polyphenols in KBT infusion were determined and are provided in Appendix A, Table A1 for reference. To evaluate the inhibitory activity of the native tea infusion, the dose–response curve of the KBT infusion was measured, yielding an IC_50_ of 13.82 ± 0.42 µg/mL, which was calculated based on the infusion prepared from 3 g tea leaves in 150 mL boiling deionized water, with a high goodness-of-fit (R^2^ = 0.9994), confirming the robustness of the assay.

For subsequent reconstruction and interaction experiments, the tea infusion was diluted 100-fold to reach an inhibition of approximately 80%, which lies within the sensitive portion of the dose–response curve. At this dilution, the concentrations of key polyphenols, including EGCG and TFDG, correspond to ranges commonly employed in food-based enzyme inhibition studies, providing a practical reference for interaction analysis. The inhibition rates of individual polyphenols were measured at the same concentrations as present in the diluted infusion. As shown in Table 1, within the esterified catechin subclass, ECG and EGCG were the most abundant at 8.14 µM and 4.34 µM, respectively, while TF and TFDG predominated among theaflavins in the diluted infusion, characterizing the unique biochemical profile of KBT formed during traditional fermentation. These single-compound values provided the baseline for calculating the theoretical additive inhibition of reconstructed mixtures, assuming no interactions between components.

The reconstructed full mixture of all eight polyphenols achieved an inhibition of 56.81 ± 2.44%, representing approximately 72% of the inhibitory activity of the diluted native KBT infusion. The remaining 28% of the inhibitory activity was not accounted for by the reconstructed system. This residual inhibition is likely attributable to minor phenolics or higher-order interactions beyond the eight reconstructed compounds. Previous studies have reported that compounds such as theasinensin A, a dimer formed during low-temperature fermentation, can contribute significantly to α-glucosidase inhibition in certain black tea varieties [6]. Although the cultivar (Zhu Ye Zhong) and traditional fermentation process of KBT differ from those in other teas, it is reasonable to speculate that theasinensin A and other minor phenolics may underlie the residual activity observed here.

These results support the conclusion that the eight selected polyphenols constitute the core bioactive module of KBT, while also highlighting the presence of additional minor constituents or interactions that contribute to the full inhibitory profile. Future inclusion of polymerized catechins may refine the model.

In addition to compositional coverage, the experimental representativeness of the reconstructed system is also influenced by how tea infusion is produced. Tea brewing is inherently variable, with factors such as leaf-to-water ratio, infusion time, temperature, and subsequent dilution influencing the absolute concentrations and relative proportions of extracted polyphenols. In the present study, a standardized household brewing protocol was adopted as a reference condition to ensure experimental consistency and relevance. Systematic evaluation of brewing variability and its impact on polyphenol interaction profiles remains an important direction for future studies aiming to bridge laboratory assays with real-world consumption scenarios.

### 3.2. Antagonistic Interactions and Net Contributions of Major Polyphenols

**Figure 2:**
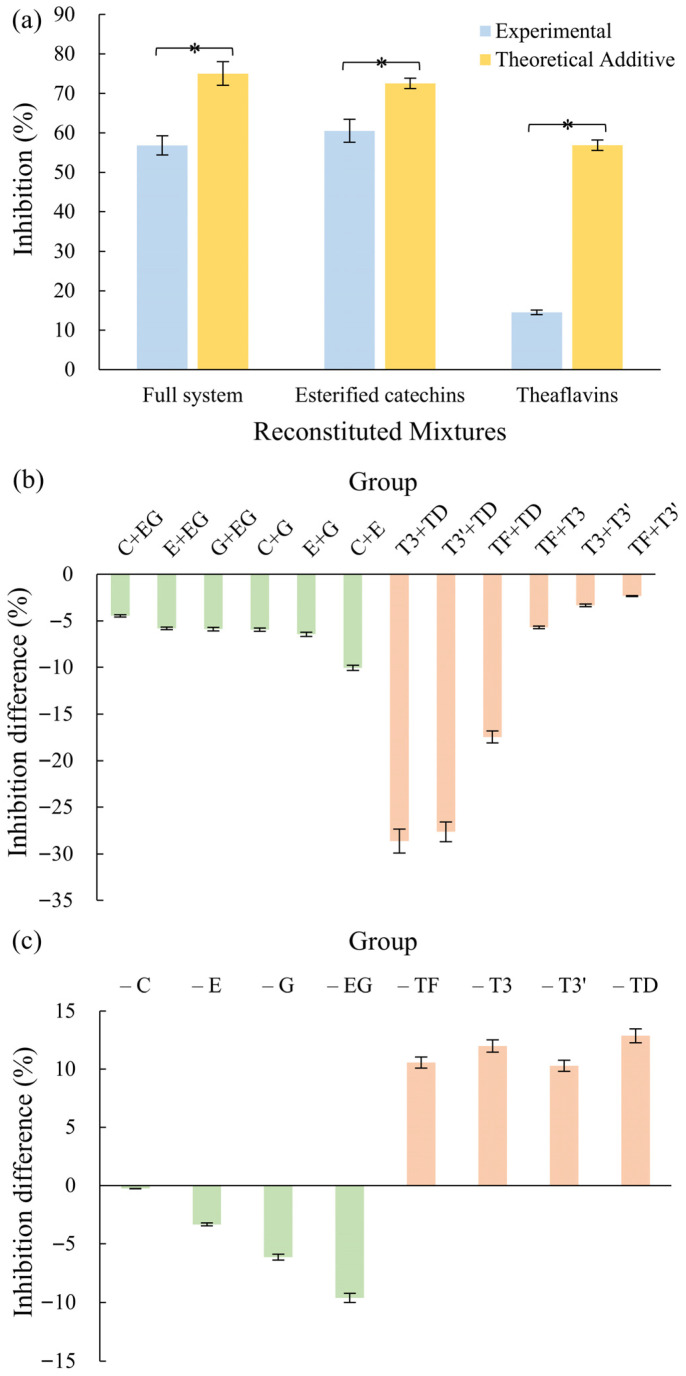
Antagonistic interactions and net contributions of Keemun black tea polyphenols: (**a**) Measured inhibition versus theoretical additive inhibition for the full system and major subclasses; (**b**) Pairwise interaction outcomes among esterified catechins and theaflavins, expressed as inhibition difference; (**c**) Net contributions of individual polyphenols revealed by systematic omission, expressed as inhibition difference. *: a statistically significant difference in panel (**a**) (*p* < 0.05). For panels (**b**,**c**), inhibition difference is defined as the difference between experimentally measured inhibition and the corresponding theoretical additive inhibition. (C = CG, E = ECG, G = GCG, EG = EGCG; T = TF, T3 = TF3G, T3′ = TF3′G, TD = TFDG).

Analysis of the reconstructed Keemun black tea (KBT) system revealed that antagonistic interactions broadly shape its α-glucosidase inhibitory activity. Comparison of the measured inhibition rates with theoretical additive values indicated a systematic deviation toward lower activity (Figure 2a), suggesting that inhibitory effects cannot be predicted by simple summation of individual compounds. This pervasive antagonism occurred both between subclasses (esterified catechins vs. theaflavins) and within each subclass, reflecting complex interplay among polyphenols under consumption-relevant conditions, seen Figure 2b. Such interactions may arise from competitive binding to overlapping enzyme active sites, as well as physicochemical factors including π–π stacking, self-association, or transient aggregation [33,34,35], which could reduce the effective concentration of free inhibitors.

Pairwise analysis (Figure 2b) confirmed that antagonism was generally dominant, with particularly strong effects observed within the theaflavin subclass. This trend likely reflects the structural features of theaflavins, such as multiple galloyl groups, which can create steric hindrance [36] or competitive exclusion when multiple high-affinity molecules coexist. The magnitude of antagonism was also concentration-dependent: pairs containing abundant compounds such as ECG and TFDG exhibited stronger antagonistic effects, indicating that high local densities of potent ligands may constrain overall inhibitory efficiency. Under the tested conditions, no visible precipitation or turbidity was observed, suggesting that these effects primarily arise from molecular interactions rather than macroscopic solubility changes.

Systematic omission experiments further delineated the net contributions of each polyphenol, as shown in Figure 2c. Removal of esterified catechins significantly reduced inhibition, indicating a net positive contribution derived from their intrinsic potency and potential but limited synergistic interactions, coupled with relatively weaker internal antagonism. In contrast, omitting theaflavins paradoxically increased overall inhibitory activity, highlighting their net negative contribution under consumption-relevant conditions. This counterintuitive outcome underscores the impact of internal antagonism, potential self-association, and competitive interactions that limit their effective inhibition in the presence of other polyphenols.

Overall, these results demonstrate that the α-glucosidase inhibitory activity of KBT emerges from a network of concentration- and structure-dependent antagonistic interactions, rather than the additive effects of individual compounds. These findings suggest that the inhibitory contribution of high-abundance polyphenols may be limited under typical tea consumption. Accordingly, the relative proportions of polyphenols are likely more influential than their absolute concentrations. It should be noted that these observations are derived from in vitro experiments; the actual effects in vivo will be modulated by digestion, absorption, and metabolism. Therefore, these results primarily provide mechanistic insights and a foundational clue for further studies using simulated gastrointestinal conditions or in vivo models to assess real-world α-glucosidase inhibition.

### 3.3. EGCG-Centered Pairwise Interactions

EGCG was identified as the most influential esterified catechin, as its removal caused the greatest reduction in inhibitory activity, and was therefore selected for pairwise analysis with theaflavins. The IC_50_ values and goodness-of-fit (R^2^) for EGCG and theaflavins are provided in Appendix A, Table A2. For each pair, the exact concentration ranges tested were defined based on their respective IC_50_ values and covered effect levels (*Fa*) from approximately 0.1 to 0.9, with concentration combinations designed to span low to high inhibition.

**Figure 3:**
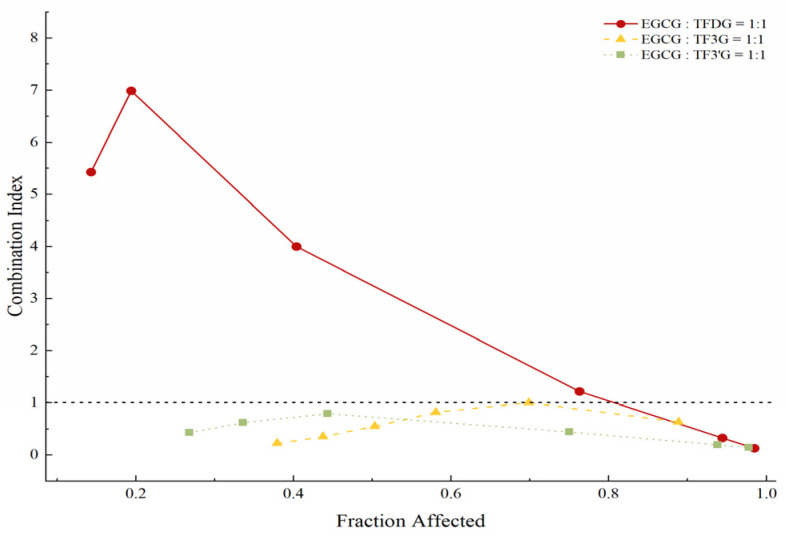
Combination index (CI) as a function of the fraction affected (*Fa*) for mixtures of representative black tea polyphenols.

As shown in Figure 3, the EGCG–TFDG pair (1:1 molar ratio) exhibited a concentration-dependent biphasic interaction: antagonism predominated at low concentrations, while synergism emerged at higher levels. EGCG also showed synergistic interactions with TF3G and TF3′G, but these did not dominate the reconstructed system. TF exhibited approximately additive effects at specific effect levels (*Fa* = 0.45 and 0.59; CI = 0.97 and 1.00). Taken together, at consumption-relevant concentrations, the strong EGCG–TFDG antagonism outweighs synergistic effects, dictating the net antagonistic nature of the inter-subclass relationship.

These results highlight the dynamic and context-dependent nature of polyphenol interactions, demonstrating that the inhibitory activity of individual compounds is modulated by the surrounding chemical environment.

### 3.4. Molecular Basis of Antagonistic Interactions

**Figure 4:**
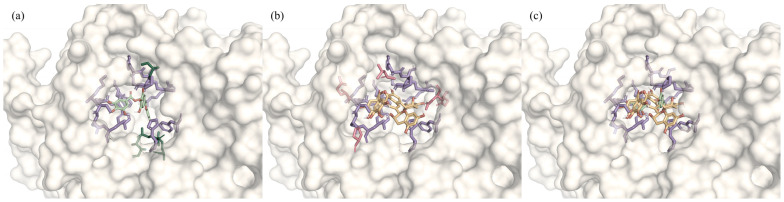
Structural basis underlying antagonistic interactions between EGCG and TFDG in α-glucosidase inhibition. (**a**) Predicted binding mode of EGCG (green) within pocket C1 of α-glucosidase, highlighting residues shared with TFDG (purple) and additional peripheral residues unique to EGCG (dark green); (**b**) predicted binding mode of TFDG (yellow), showing shared residues (purple) and additional peripheral residues unique to TFDG (rose); (**c**) overlay of EGCG and TFDG within pocket C1 of α-glucosidase, highlighting the conserved set of shared residues (purple).

To elucidate the structural basis of the observed antagonism, molecular docking of the eight major polyphenols with α-glucosidase was performed. All compounds preferentially occupied the primary binding pocket (C1) of α-glucosidase, indicating strong competition for a common binding site (Table 2). Overlaying EGCG and TFDG within pocket C1 revealed substantial spatial overlap (Figure 4), with both ligands sharing most key residues, including LYS156, TYR158, PHE159, HIS280, and ASP352. This extensive overlap provides a structural basis for the antagonism observed at consumption-relevant concentrations.

Although both compounds occupy the same pocket, they adopt distinct binding strategies. EGCG interacts primarily through a distributed hydrogen-bonding network with peripheral residues such as ASP69 and ASP215, a flexible mode typical of polyhydroxylated catechins that renders it susceptible to displacement. In contrast, TFDG binds more centrally and deeply within the pocket, stabilized by π–π stacking with aromatic residues including TRP238 and TRP326, resulting in more rigid anchoring and lower Vina scores (Table 2). These differences provide a plausible molecular explanation for the concentration- and structure-dependent antagonism observed experimentally.

Together, these molecular insights support the experimental findings, showing that multiple high-affinity ligands in a shared pocket generate competition-driven antagonism rather than additive inhibition.

## 4. Conclusions

This study demonstrates that, under consumption-relevant conditions, the α-glucosidase inhibitory activity of Keemun black tea is governed by concentration- and structure-dependent interactions among its major polyphenols, rather than the simple additive effects of individual constituents. Despite their strong individual potencies, theaflavins contributed negatively to overall inhibition under consumption-relevant conditions due to pronounced intra- and interclass antagonism, whereas esterified catechins—particularly EGCG—provided net positive contributions, reflecting their higher relative abundance and weaker internal antagonism.

CI analysis revealed that these interactions are highly concentration-dependent, and molecular docking confirmed extensive binding-site overlap on α-glucosidase, offering a structural explanation for competitive exclusion among coexisting polyphenols. These findings highlight the importance of adopting a system-level perspective for understanding bioactivity in chemically realistic food matrices, beyond reductionist, single-compound approaches.

Future studies should extend these findings to more physiologically relevant contexts, including simulated gastrointestinal digestion, gut microbiota-mediated metabolism, and mammalian enzyme systems, to evaluate the consistency of interaction patterns beyond in vitro screening models. In addition, examining other tea varieties and multi-component beverages will help determine which aspects of the observed interaction behavior are system-specific and which may reflect more general principles of polyphenol coexistence. Such efforts will further clarify how relative polyphenol composition and concentration-dependent interactions influence real-world α-glucosidase inhibition and support the development of more predictive frameworks for assessing bioactivity in complex dietary matrices.

## Figures and Tables

**Table 1:** Concentrations and inhibition of individual polyphenols in the 100-fold diluted Keemun black tea infusion.

Compound Subclass	Polyphenol Monomers	Concentration (µM) ^1^	Inhibition (%)
Esterified Catechins	Catechin Gallate, CG	1.627 ± 0.029	10.32 ± 0.37
	Epicatechin Gallate, ECG	8.143 ± 0.045	17.17 ± 0.66
	Gallocatechin gallate, GCG	2.635 ± 0.050	19.41 ± 0.52
	Epigallocatechin Gallate, EGCG	4.335 ± 0.017	25.64 ± 0.95
Theaflavins	Theaflavin, TF	0.345 ± 0.005	2.79 ± 0.05
	Theaflavin-3-gallate, TF3G	0.106 ± 0.003	16.12 ± 0.63
	Theaflavin-3′-gallate, TF3′G	0.039 ± 0.006	5.78 ± 0.18
	Theaflavin-3,3′-digallate, TFDG	0.245 ± 0.003	32.17 ± 1.16

^1^ Calculated based on the molecular weight of each monomer for pairwise molar ratio experiments.

**Table 2:** Molecular docking of esterified catechins and theaflavins with α-glucosidase.

Compounds		Binding Pocket	Vina Score (kcal/mol)	Shared Key Residues	Class-Specific Residues
Esterified catechins	CG	C1	−10.5	LYS156, TYR158, PHE159, GLY160, PHE178, GLN239, ASP242, HIS280, PHE303, THR306, ASP307, ARG315, TYR316, ASP352, GLN353, GLU411, ASN415, ARG442	ASP69, ASP215
	ECG		−9.8		
	GCG		−10.1		
	EGCG		−10.1		
Theaflavins	TF	C1	−10.3	LYS156, TYR158, PHE159, GLY160, PHE178, GLN239, ASP242, HIS280, PHE303, THR306, ASP307, ARG315, TYR316, ASP352, GLN353, GLU411, ASN415, ARG442	TRP238, GLU332
	TF3G		−11.1		
	TF3′G		−11.1		
	TFDG		−11.7		

## Data Availability

The original contributions presented in the study are included in the article. Further inquiries can be directed to the corresponding authors.

## Abbreviations

The following abbreviations are used in this manuscript:

KBT	Keemun black tea
CI	Combination Index
CG	(-)-catechin gallate
GCG	(-)-gallocatechin gallate
ECG	(-)-epicatechin gallate
EGCG	(-)-epigallocatechin-3-gallate
TF	theaflavin
TF3G	theaflavin-3-gallate
TF3′G	theaflavin-3′-gallate
TFDG	theaflavin-3,3′-digallate
T2DM	type 2 diabetes mellitus
pNPG	p-nitrophenyl-α-D-glucopyranoside
R^2^	the coefficient of determination

## Appendix A

**Table A1:** Concentrations of the eight target polyphenols in Keemun black tea infusion.

Compound Subclass	Polyphenol Monomers	Concentration (mg/mL) ^1^
Esterified Catechins	Catechin Gallate, CG	0.0720 ± 0.0013
	Epicatechin Gallate, ECG	0.3602 ± 0.0020
	Gallocatechin gallate, GCG	0.1208 ± 0.0023
	Epigallocatechin Gallate, EGCG	0.1987 ± 0.0008
Theaflavins	Theaflavin, TF	0.0195 ± 0.0003
	Theaflavin-3-gallate, TF3G	0.0076 ± 0.0002
	Theaflavin-3′-gallate, TF3′G	0.0028 ± 0.0004
	Theaflavin-3,3′-digallate, TFDG	0.0213 ± 0.0003

^1^ Calculated through the external standard curve with R^2^ > 0.999, and expressed as mean ± standard deviation (*n* = 3).

**Table A2:** Half maximal inhibitory concentration (IC_50_) values and dose–response fitting parameters of selected α-glucosidase inhibitors.

Inhibitor	IC_50_	R^2^
Epigallocatechin Gallate, EGCG	6.69 ± 0.14 μM	0.9999
Theaflavin, TF	21.23 ± 0.88 μM	0.9997
Theaflavin-3-gallate, TF3G	1.51 ± 0.25 μM	0.9909
Theaflavin-3′-gallate, TF3′G	1.64 ± 0.04 μM	0.9998
Theaflavin-3,3′-digallate, TFDG	0.86 ± 0.07 μM	0.9979
Acarbose	10.08 ± 0.75 mM	0.9999
